# Using a Single Measure To Assess Adherence and Differentiation in Family Therapy for Adolescent Externalizing Problems

**DOI:** 10.1007/s10488-025-01445-y

**Published:** 2025-04-28

**Authors:** Stephanie Violante, Bryce D. McLeod, Aaron Hogue

**Affiliations:** 1 Department of Psychology, Virginia Commonwealth University, VA Richmond, USA; 2 Partnership to End Addiction, Family and Adolescent Clinical Technology & Science, New York, NY USA

**Keywords:** Treatment integrity, Family therapy, Adolescents, Externalizing problems

## Abstract

The interpretation of effectiveness research can be enhanced by understanding what prescribed (i.e., adherence) and non-prescribed (i.e., differentiation) techniques were delivered. However, few measures exist that can assess both adherence and differentiation. The current study examined how the Therapy Process Observational Coding System for Child Psychotherapy Revised Strategies Scale (TPOCS-RS) can assess adherence to and differentiation from family therapy for youth with externalizing problems. Treatment sessions (*N* = 103) from 42 adolescents (*M* age = 15.0, *SD* = 1.4; 47.6% female; 59.5% Hispanic/Latinx/e, 19.0% Black, 11.9% multiracial, 4.8% other race) with primary externalizing problems treated by 24 clinicians (*M* age = 33.2, *SD* = 8.3; 66.7% female; 33.3% Hispanic/Latinx/e, 20.8% White, 12.5% Asian, 8.3% multiracial, 8.3% other race) in routine practice settings were coded with the TPOCS-RS. Treatment sessions were from three groups: (a) routine family therapy, (b) routine family therapy plus the medication integration protocol, or (c) usual care. Interrater reliability for the TPOCS-RS Family Therapy subscale was ICC = 0.90, and scores demonstrated evidence of convergent and discriminant validity via associations with treatment integrity and alliance measures. The TPOCS-RS Family Therapy subscale also demonstrated evidence of discriminative validity by identifying expected group differences. Results provide preliminary evidence that the TPOCS-RS can measure adherence to and differentiation from family therapy.

**Supplementary Information:**

The online version contains supplementary material available at 10.1007/s10488-025-01445-y.

The ability to interpret results from effectiveness research requires measuring treatment integrity. Treatment integrity is defined as the degree to which an intervention is delivered as intended (Allen et al., 2012; McLeod et al., 2013). While there is no single agreed-upon conceptualization of treatment integrity, in effectiveness research, treatment integrity commonly is considered to comprise adherence (i.e., the extent to which prescribed [core] therapeutic techniques are delivered), competence (i.e., the skill and responsiveness with which prescribed techniques are delivered), and differentiation (i.e., avoiding the delivery of non-prescribed [non-core] techniques not found in a treatment protocol; Allen et al., 2012; McLeod et al., 2013). Measurement of treatment integrity most often focuses on adherence and, to a lesser extent, differentiation (Cox et al., 2019). However, assessing adherence and differentiation may be important for effectiveness research (Sutherland et al., 2022).

Effectiveness research often produces mixed findings (Weisz et al., 2013). The measurement of adherence can aid the interpretation of effectiveness research. Adherence measurement allows researchers to perform a manipulation check by determining if levels of adherence in the treatment group were sufficient. The measurement of adherence can also help researchers ascertain if there was contamination bias in usual clinical care (Schoenwald et al., 2011). Usual clinical care and evidence-based programs may have overlapping techniques (Borntrager et al., 2013; Smith et al., 2017), contributing to the smaller effects observed in effectiveness research. Measuring adherence across groups in an effectiveness trial thus provides information that allows researchers to interpret study findings, whether they favor the treatment or usual clinical care group (Schoenwald et al., 2011).

When measured concurrently with adherence, differentiation can help researchers interpret findings from effectiveness research. Clinicians in effectiveness research have varied training backgrounds and may deliver various techniques from different treatment modalities (Garland et al., 2010; Smith et al., 2017). Thus, many techniques delivered by clinicians in the treatment and usual clinical care groups are likely not from the treatment protocol (Garland et al., 2014). Assessing differentiation can help determine what techniques from outside the treatment protocol were delivered in the treatment group and usual clinical care. If usual clinical care is effective, differentiation assessment (i.e., determining what non-prescribed techniques were delivered in both treatment groups) can represent a source of practice-based evidence (Garland et al., 2014).

Though assessing adherence and differentiation in effectiveness research has benefits, most treatment integrity measures assess only one integrity component (Cox et al., 2019). Thus, capturing adherence and differentiation can require multiple measures. However, using a single measure to capture adherence and differentiation could be more efficient and facilitate cross-group comparisons (Schoenwald et al., 2011). Overall, there are several practical benefits to a measure that can be used to assess adherence and differentiation.

The Therapy Process Observational Coding System for Child Psychotherapy Revised Strategies Scale (TPOCS-RS; McLeod et al., 2015; McLeod et al., 2022) is an observational measure that captures a wide array of techniques and has the potential for assessing differentiation and adherence. The TPOCS-RS assesses how extensively a clinician delivers discrete techniques (e.g., cognitive education, relaxation) found across five treatment orientations for youth emotional and behavioral problems: cognitive, behavioral, family, client-centered, and psychodynamic. Because the TPOCS-RS is not specific to a clinical problem (e.g., anxiety, depression) or treatment approach (e.g., cognitive-behavioral therapy), it may be well-suited to assess differentiation in effectiveness research (McLeod et al., 2013, 2015).

Existing evidence supports the use of the TPOCS-RS as a measure of differentiation. First, McLeod et al. (2015) found that the magnitude of the correlations among the five subscales (i.e., Cognitive, Behavioral, Family, Client-Centered, and Psychodynamic) were small to medium, providing evidence that the subscales demonstrate discriminant validity and thus measure distinct techniques. Further, Smith et al. (2017) found that clinicians delivering manual-based cognitive-behavioral therapy (CBT) in research and practice settings delivered the highest dosage of cognitive-behavioral techniques, followed by client-centered, family, and psychodynamic. In contrast, usual care clinicians delivered the highest dosage of family techniques, followed by client-centered, psychodynamic, and cognitive-behavioral. Overall, evidence suggests that the TPOCS-RS can assess CBT and non-CBT (e.g., psychodynamic) and discriminate between treatment groups (CBT vs. usual clinical care), supporting the use of the TPOCS-RS as a measure of differentiation in effectiveness research focused on CBT.

Although the TPOCS-RS was not originally designed to assess adherence to a specific treatment protocol, its design features may allow it to be used as such. Recent evidence suggests that the TPOCS-RS can estimate adherence to a CBT protocol for youth anxiety (McLeod et al., 2022). In a sample of youth seeking treatment for anxiety problems receiving a standard manualized CBT program, a modular manualized CBT program, or usual clinical care, items on the TPOCS-RS Anxiety subscale comprised of techniques found in CBT programs had excellent interrater reliability. The Anxiety items and subscale also displayed evidence of convergent and discriminant validity when used to estimate protocol adherence to two distinct CBT protocols for youth anxiety. Specifically, the TPOCS-RS Anxiety subscale correlated more highly with two protocol-specific adherence measures than the TPOCS-RS Psychodynamic, Family, and Client-Centered subscales (McLeod et al., 2022). Overall, the TPOCS-RS scores displayed evidence of reliability and construct validity when used to estimate protocol adherence to two manualized CBT programs, indicating that the TPOCS-RS may approximate protocol adherence for CBT.

Though evidence suggests that the TPOCS-RS may be suitable for measuring adherence and differentiation, this evidence is limited to samples of youth receiving CBT. To enhance the value of the TPOCS-RS for effectiveness research, it is essential to examine the evidence for the construct validity of other subscales. This study aims to assess the TPOCS-RS’s ability to measure adherence to and differentiation from structural-strategic family therapy (hereafter called *family therapy*). Family therapy is a treatment modality that intervenes directly with family members to address dysfunctional interactions and challenges within the family system that contribute to the onset and maintenance of adolescent behavior problems (Baldwin et al., 2012; Hogue et al., 2019). This treatment approach has strong empirical support for treating adolescent externalizing problems, including conduct problems, delinquency, and substance misuse (Baldwin et al., 2012). Demonstrating that the TPOCS-RS can assess treatment adherence to and differentiation from the family therapy modality will broaden the utility of the measure for effectiveness research.

To demonstrate that the TPOCS-RS items and subscales can assess adherence to and differentiation from family therapy, the TPOCS-RS should display specific psychometric characteristics. First, items that capture techniques found in family therapy should demonstrate adequate interrater reliability (i.e., intraclass correlation [ICC] *≥* 0.40; Cicchetti, 1994) when used to code treatment sessions containing family therapy (Carroll et al., 2000; Hogue et al., 2008). Second, to provide evidence that scores can be used to assess adherence to family therapy, TPOCS-RS items combined into a Family Therapy subscale should display evidence of convergent validity by demonstrating strong associations with measures designed to assess adherence to family therapy (McLeod et al., 2022). Third, to provide evidence that scores can be used to assess differentiation (i.e., the scores do not overlap with other modality-based domains), the TPOCS-RS items combined in a Family Therapy subscale should display weaker associations (discriminant validity) with (a) measures designed to assess other therapy domains (i.e., Behavioral Parent Training [BPT], CBT, Client-Centered, Psychodynamic), and (b) measures of the client-clinician alliance (McLeod et al., 2022). To be helpful to researchers in interpreting results from effectiveness research and evaluating differentiation, the TPOCS-RS should be able to discriminate between treatment groups expected to differ in their delivery of therapeutic techniques, in this case, groups delivering family therapy from a group not delivering family therapy (i.e., discriminative validity; Southam-Gerow et al., 2016). Finally, to demonstrate that the TPOCS-RS can assess adherence and differentiation, the Family Therapy subscale should be relatively high for groups delivering family therapy whereas the subscales that assess techniques non-prescribed in family therapy (e.g., CBT, BPT, Psychodynamic) should be relatively low, and consistent across the treatment groups (McLeod et al., 2013).

To achieve study goals, the TPOCS-RS was used to code treatment sessions from adolescents receiving treatment for externalizing problems assigned to one of three groups: routine family therapy (RFT), family therapy plus the medication integration protocol (MIP), or usual clinical care (UC). In the current study, convergent and discriminant validity of scores on the TPOCS-RS Family Therapy subscale are supported if the following pattern of correlations is observed: (a) correlations are highest with a measure designed to assess adherence to family therapy (Cecilione et al., 2021; Southam-Gerow et al., 2016), and (b) correlations are lower with subscales and measures purported to assess other therapy domains (e.g., subscales that assess non-family therapy [behavioral parent training], CBT) and the client-clinician alliance (Carroll et al., 2000; Hogue et al., 2008). Discriminative validity in the current study will be supported if the TPOCS-RS Family Therapy subscale scores are highest for the groups known to contain family therapy and consistent with integrity scores found in past research (Hogue et al., 2014b, 2016). Finally, differentiation in the current study will be supported if the TPOCS-RS subscales non-prescribed by family therapy (e.g., CBT, BPT, Psychodynamic) are low in dosage and also similar across groups.

## Method

### Data Sources

The current study leverages archived data from a randomized naturalistic trial (CASALEAP; Hogue et al., 2015a) and a pilot study (CASALEAP-MIP, hereafter referred to as (MIP; Hogue et al., 2016). The CASALEAP trial compared family therapy to nonfamily therapy for adolescents with externalizing problems in routine practice settings. Youth with externalizing problems, including disruptive or delinquent behavior and substance use, were clinically referred to the research staff by high schools, family service agencies, and community programs serving youth in urban areas of a large northeastern city. Adolescents were randomly assigned to one of two groups: (a) Routine Family Therapy (RFT) or (b) Usual Care (UC). Adolescents assigned to RFT received treatment at a single community mental health clinic that routinely delivered non-manualized family therapy. Adolescents assigned to UC received treatment at one of five sites that did not routinely deliver family therapy. Youth assigned to RFT experienced greater reductions in youth-reported externalizing and internalizing symptoms, delinquency, and alcohol and drug use than youth in the UC group (Hogue et al., 2015a).

The MIP trial evaluated a family-based protocol designed to integrate medication services into psychosocial treatment planning for adolescents referred for primary externalizing problems with comorbid attention-deficit/hyperactivity disorder (ADHD). Youth were clinically referred and received treatment at a community mental health clinic that provided family therapy as the standard of care for youth externalizing problems (the same clinic that treated RFT cases in the CASALEAP trial). Youth in the MIP trial were compared with a matched historical control group, which comprised adolescents who were assigned to RFT in the CASALEAP trial and met diagnostic criteria for ADHD. Youth in MIP were found to be more likely to complete a psychiatric evaluation and initiate medication for ADHD than youth in the historical control group (Hogue et al., 2016). The parent studies and the current study all received institutional review board approval.

## Participants

**CASALEAP.** Two hundred and five youth (*M* age = 15.7 years, *SD* = 1.5; 48% female, 52% male; 59% Hispanic/Latinx/e, 21% Black, 15% multiracial, 6% other race) were enrolled in the CASALEAP trial. Youth were included if they (a) were between 12 and 18 years old; (b) had a primary caregiver who was willing to participate in treatment; (c) met criteria for either the Mental Health or Substance Use track; (d) were not enrolled in any other psychosocial treatment; (e) expressed willingness to participate in treatment; and (f) had a health insurance plan that was accepted at the study treatment sites. Youth were excluded if they (a) had an intellectual disability or autism spectrum disorder, (b) had a medical or psychiatric illness requiring hospitalization, (c) had current psychotic symptoms, or (d) had active suicidal ideation. Thirty youths from the CASALEAP trial were included in the current study. Included youth were a subsample of CASALEAP participants with available video recordings based on a sample of videorecorded sessions randomly sampled from early and late treatment. Included youths averaged 14.6 years old (*SD* = 1.3), were 50.0% female and 50% male, and identified as 66.7% Hispanic/Latinx/e, 13.3% Black, 13.3% Multiracial, and 6.7% other race. 63% of participants had a primary caregiver with a yearly income of over $15,000. See Table 1 for details.

**Table 1:** Youth descriptive data and group comparisons

	M (SD) or %			*F* or *χ2 *(*p-*value)
	RFT (*n* = 16)	UC (*n* = 14)	MIP (*n* = 12)	
Age	14.45 (1.37)	14.78 (1.23)	16.30 (1.04)	7.25 (0.002)
Sex	–	–	–	0.00 (1.00)
Female	50.0	50.0	41.7	–
Male	50.0	50.0	41.7	–
NR	0.0	0.0	16.7	–
Race/Ethnicity	–	–	–	5.01 (0.543)
Latinx/e	75.0	57.1	41.7	–
Black	6.3	21.4	33.3	–
Multiracial	12.5	14.3	8.3	–
Other race	6.3	7.1	0.0	–
NR	0.0	0.0	16.7	–
Income > 15k	50.0	78.4	–	2.63 (0.142)

Note. UC = usual care; RFT = routine family therapy; MIP = medication integration protocol; NR = not reported

A total of 34 clinicians (*M* age = 32.7 years, *SD* = 8.1; *M* years experience = 3.1, *SD* = 3.2; 58.8% female, 20.6% male, 20.6% not reported; 38.2% White, 26.5% Hispanic/Latinx/e, 8.8% Asian, 5.9% other race, 20.6% not reported) participated in the CASALEAP trial. Clinicians in the RFT group (*n* = 14) were from a community mental health clinic that provided family therapy as the standard approach. All clinicians received regular training and supervision from on-site supervisors experienced in family therapy. Clinicians in the UC group (*n* = 20) were from one of five outpatient clinics that did not routinely deliver family therapy. None of the UC clinicians or supervisors were marriage and family clinicians or completed postgraduate training in family therapy. The current study included a total of 21 CASALEAP clinicians (*M* age = 33.7 years; *SD* = 8.9; 61.9% female, 38.1% male) who averaged 3.2 years of post-degree therapy experience (*SD* = 3.5) and identified as 28.6% Hispanic/Latinx/e, 19.0% White, 14.3% Asian American, 9.5% Multiracial, and 9.5% other race. CASALEAP clinicians self-reported allegiance to and skill in family therapy (allegiance *M* = 3.3, *SD* = 1.2; skill *M* = 3.0, *SD* = 1.0), CBT (allegiance *M* = 2.8, *SD* = 1.0; skill *M* = 3.1, *SD* = 0.8), and motivational interviewing (allegiance *M* = 2.2, *SD* = 1.2; skill *M* = 2.5, *SD* = 1.0) rated on a scale from 1 to 5 where 1 = *none*, 3 = *moderate*, and 5 = *high*. See Table 2 for details.

**Table 2:** Clinician descriptive data and group comparisons

	M (SD) or %			*F*, *t*, or *χ2 *(*p-*value)
	RFT (*N* = 12)	UC (*N* = 9)	MIP (*N* = 3)	
Age	37.4 (11.5)	30.3 (4.1)	30.3 (1.5)	1.90 (0.180)
Sex	–	–	–	2.03 (0.362)
Female	58.3	66.7	100	–
Male	8.3	33.3	0.0	–
NR	33.3	0.0	0.0	–
Race/Ethnicity	–	–	–	8.65 (0.373)
Hispanic/Latinx/e	41.7	11.1	66.6	–
White	8.3	33.3	33.3	–
Asian	0.0	33.3	0.0	
Multiracial	83	11.1	0.0	–
Other race	8.3	11.1	0.0	–
NR	33.3	0.0	0.0	–
Years experience	3.7 (4.8)	2.9 (2.5)	3.0 (1.7)	0.98 (0.907)
Allegiance	–	–	–	–
Family therapy	3.6 (1.3)	3.0 (1.0)	NR	1.12 (0.281)
CBT	2.4 (1.1)	3.2 (0.8)	NR	−1.84 (0.085)
MI	2.3 (1.3)	2.1 (1.2)	NR	0.23 (0.818)
Skills	–	–	–	–
Family therapy	3.5 (0.8)	2.6 (1.0)	NR	2.15 (0.048)*
CBT	2.8 (0.9)	3.3 (0.5)	NR	−1.70 (0.110)
MI	2.6 (1.1)	2.4 (1.0)	NR	0.359 (0.725)

Note. UC = usual care; RFT = routine family therapy; MIP = medication integration protocol; NR = not reported; years experience = years post-degree therapy experience; allegiance = self-reported allegiance to therapeutic approach; skills = self-reported skills in therapeutic approach; CBT = cognitive-behavioral therapy; MI = motivational interviewing

*p <.05

**Medication Integration Protocol.** Thirty-five adolescents (*M* age = 15.3, *SD* = 1.3; 50.0% female, 50.0% male) participated in MIP. Inclusion criteria included: (a) age 13 to 17; (b) a primary caregiver who was willing to participate in treatment; (c) met DSM-IV diagnostic criteria for ODD, CD, or substance use disorder (SUD); (d) met DSM-IV criteria for ADHD with or without onset prior to age 7; (e) were not prescribed medication for ADHD; and (f) were not enrolled in any behavioral treatment. Exclusion criteria included (a) bipolar disorder, (b) intellectual disability, (c) pervasive developmental disorder, (d) medical or psychiatric illness requiring hospitalization, (e) current psychotic symptoms, and (f) current suicidal ideation. Twelve youths from the MIP trial were included in the current study. Included youths were a subsample of MIP participants with available video recordings based on a sample of videorecorded sessions randomly sampled from early and late treatment. Included youths averaged 16.30 years old (*SD* = 1.04), were 41.7% female and 58.3% male, and identified as 41.7% Hispanic/Latinx/e, 33.3% Black, and 8.3% Multiracial. Eleven clinicians participated in the MIP trial, all from a single community mental health clinic that provided family therapy as the standard of care. The current study included all three clinicians delivering MIP (*M* age = 30.30 years, *SD* = 1.50; *M* years experience = 3.00, *SD* = 1.70, 100% female; 66.6% Hispanic/Latinx/e, 33.3% White). MIP clinicians did not rate their allegiance to and skill in different treatment modalities. See Tables 1 and 2 for details.

## Treatment Groups

### CASALEAP

Across treatment groups, clinicians were not provided with external training or financial support and were not required to adjust their usual clinical practices.

**Routine Family Therapy.** The RFT group consisted of one community mental health clinic that routinely delivered family therapy. Hogue and Dauber (2013) found that the clinicians delivering family therapy adhered to gold-standard levels of adherence. Previous research on the CASALEAP sample found that RFT clinicians reported the strongest allegiance and skill in family therapy techniques (Hogue et al., 2014b). Further, RFT clinicians reported utilizing family therapy techniques more than CBT, motivational interviewing (MI), or drug counseling (DC; Hogue et al., 2014b).

**Usual Care.** The UC group consisted of five clinics representing an array of outpatient treatment settings. None of the five clinics included staff clinicians or supervisors trained or experienced in family therapy. Previous research on this study sample revealed that UC clinicians reported the strongest allegiance and skill in CBT and MI, and UC clinicians reported greater use of CBT, MI, and DC. RFT clinicians reported greater use of family therapy techniques than UC clinicians (Hogue et al., 2014b).

## Medication Integration Protocol

Participants in the Medication Integration Protocol (MIP) group received treatment at a single community-based mental health clinic that featured family therapy as their routine standard of care for adolescent externalizing problems. In addition to receiving family therapy, MIP (Hogue et al., 2014a) was delivered, which is a modular family-based protocol that integrates medication services into psychosocial treatment for adolescents with ADHD. The protocol contains five MIP Tasks, four of which can be delivered in the clinically indicated order (Task 1 always occurs first). The tasks are (1) *ADHD Assessment and Medication Consult*; (2) *ADHD Psychoeducation and Client Acceptance*; (3) *ADHD Symptoms and Family Relations;* (4) *Medication and Family Decision Making*; and (5) *Medication Management and Integration Planning*. (For a more detailed description of the MIP, see Hogue et al., 2014a).

### Measures

**Therapy Process Observational Coding System for Child Psychotherapy Revised Strategies Scale (TPOCS-RS;McLeod et al.,**^2015^**; McLeod et al.,**^2022^**)**. The TPOCS-RS is a 47-item observational measure designed to assess the delivery of therapeutic techniques across five modality-based subscales: Family (8 items; e.g., “Multiparticipant Interaction”), Cognitive (4 items; e.g., “Cognitive Distortion”), Behavioral (9 items; e.g., “Behavioral Activation”), Psychodynamic (5 items; e.g., “Interpretation”), and Client-Centered (4 items; e.g., “Positive Regard”). The TPOCS-RS also contains 17 items representing common techniques not associated with a specific treatment modality (e.g., “Homework”). Coders rate the degree to which clinicians deliver each technique during an entire session on a 7-point extensiveness scale where 1 = *not at all*, 4 = *considerably*, and 7 = *extensively*. When coders rate extensiveness, they consider both thoroughness (the depth, complexity, or persistence with which the clinician engages in a technique) and frequency (how often the clinician delivers it). The TPOCS-RS has demonstrated evidence of interrater reliability with mean ICCs ranging from 0.67 to 0.86 (McLeod et al., 2015, 2022), convergent validity (McLeod et al., 2022), discriminant validity (McLeod & Weisz, 2010; Southam-Gerow et al., 2016), and discriminative validity for CBT and BPT (McLeod et al., 2022; Smith et al., 2017; Wood et al., 2006). The TPOCS-RS was used to code RFT, MIP, and UC sessions. Only the 30 items found across the five modality-based subscales were coded for the current study. TPOCS-RS subscales were calculated by first averaging items across coders and then averaging all items in each subscale. The ICCs for the TPOCS-RS Psychodynamic and Client-Centered subscales in the total sample were > 0.75. For the current study, a TPOCS-RS Family Therapy subscale was created using five items to assess adherence to family therapy: *Targets Others*, *Recruits Others*, *Parenting Style*, *Multiparticipant Interactions*, and *Family Roles*. The existing TPOCS-RS Psychodynamic and Client-Centered subscales were used to assess differentiation, as were newly created CBT and Behavioral Parent Training subscales. See below for more information about subscale creation.

## Measures Used for Validity Analyses

**Inventory of Therapy Techniques-Adolescent Behavior Problems (ITT-ABP;Hogue et al., **2014b**,**2015b**)**. The ITT-ABP is a 25-item measure, rated by both clinicians and independent observers, designed to capture the delivery of discrete techniques associated with family therapy, CBT, MI, and DC. Items related to family therapy (e.g., arranged, coached, and helped process interactions among family members in session) and CBT (e.g., taught client new problem-solving) were drawn from the Therapist Behavior Rating Scale (TBRS; Hogue et al., 1998) and items associated with MI (e.g., praised client’s change efforts) and DC (e.g., discussed cravings, triggers, and high-risk situations that lead to current or future drug use) were drawn from the Motivational Enhancement Therapy and Twelve Step Facilitation subscales of the Yale Adherence and Competence Scale (Carroll et al., 2000). Exploratory and confirmatory factor analysis revealed a three-factor solution, combining the CBT and MI items into a single CBT/MI subscale, a Family Therapy (FT) subscale, and a DC subscale (Hogue et al., 2014b). Due to a lack of variance in the DC subscale stemming from the inability to collect session recordings from the only clinic specializing in substance use and addiction treatment, the DC subscale was not included in analyses for the current study (Hogue et al., 2015a). The ITT-ABP assesses technique delivery’s extensiveness (i.e., thoroughness and frequency) based on a 5-point Likert-type scale: 1 = *not at all*, 2 = *a little bit*, 3 = *moderately*, 4 = *considerably*, and 5 = *extensively*. The observer-and clinician-rated versions were included in the CASALEAP trial, whereas only the observer-rated version was used in the MIP trial. Interrater reliability for independent observers was ICC = 0.87 for the FT subscale and ICC = 0.76 for the CBT/MI subscale, and there was strong agreement between clinician and independent observer ratings of the ITT-ABP FT subscale (ICC = 0.64 to ICC = 0.75; Hogue et al., 2015b, 2022). Further, the FT subscale has demonstrated evidence of convergent, discriminant, discriminative, and predictive validity (Henderson et al., 2019; Hogue et al., 2014b, 2015b). In the current sample, interrater reliability between independent observers was ICC = 0.82 for the FT subscale and ICC = 0.79 for the CBT/MI subscale. For information about ITT-ABP training and coding procedures, see Hogue et al., (2014b, 2015b).

**Vanderbilt Therapeutic Alliance Scale Revised Short Form (VTAS-R-SF;Shelef & Diamond, **2008**).** The VTAS-R-SF was used to assess client-clinician alliance in the CASALEAP and MIP trials. The VTAS-R-SF is a 5-item observer-rated measure that assesses the collaborative and task-oriented working relationship between the client and the clinician. The VTAS-R-SF is rated on a 6-point scale ranging from 0 = *not at all* to 5 = *a great deal*. The VTAS-R has demonstrated internal consistency ranging from α = 0.93 to 0.98 and interrater agreement ranging from ICC = 0.80 to 0.93 (Diamond et al., 1999). The VTAS-R-SF has also demonstrated convergence with the full-length VTAS-R (Shelef & Diamond, 2008). In the current sample, the VTAS-R demonstrated internal consistency of α = 0.81 and interrater agreement ranging from ICC = 0.68 to 0.85.

## Coding and Session Sampling Procedures

Two female clinical psychology doctoral students coded the TPOCS-RS (*M age* = 28.5, *SD* = 0.7; 100% White). Training consisted of four phases. First, coders read and discussed the scoring manual. Second, coders scored five sessions together and discussed discrepancies. Third, coders scored 10 sessions independently and discussed coding results. Fourth, 30 sessions were independently scored, and coders were required to reach adequate item-level reliability, ICC (2,2) > 0.59 (Cicchetti, 1994), before coding independently. Coders were naïve to the group from which the sessions were derived, and the order of sessions was randomly assigned to coders. During coding, coders met regularly to discuss questions. Interrater reliability was examined on an ongoing basis to ensure reliability. If item-level reliability fell below an acceptable level (ICC < 0.60) for an item, additional training occurred. Coders scored entire treatment sessions, and every session was double-coded. One hundred three sessions were coded from CASALEAP (*n* = 35 RFT, *n* = 19 UC) and MIP (*n* = 49). The sessions were randomly selected from early (sessions 1–4; 44% for CASALEAP, 65% for MIP) and late (session six or higher; 56% for CASALEAP, 35% for MIP) treatment. The average number of sessions coded per case was 2.13 (range 1–7) in RFT, 1.36 (range 1–2) in UC, and 4.45 (range 2–10) in MIP.

## Results

### Preliminary Analyses

Sample bias analyses were run to determine if the youths and clinicians included in this study differed from those in the parent studies. The only difference was that youth in this sample were younger than the CASALEAP youth not included in the study, *t*(203) = 4.50, *p* <.001. No differences were found for clinicians. We also compared youth and clinicians across the three groups used in this study. For the youth included in this study, comparisons revealed significant differences in age across the three groups (*F*[2, 37] = 7.25, *p* =.002). For the clinicians included in the current study, those in the RFT group self-reported a higher level of skill in RFT (*M* = 3.5, *SD* = 0.8) than those in the UC group (*M* = 2.6, *SD* = 1.0, *t*(15) = 2.15, *p* =.048). See Tables 1 and 2.

### Interrater Reliability

Interrater reliability was examined using ICCs. The model ICC (2,2) based on a two-way random effects model was used as it provides a reliability estimate of the mean score of multiple coders and allows for the generalization of findings to other samples (Koo & Li, 2016). Following the recommendations by Cicchetti (1994), ICCs below 0.40 are *poor*, between 0.40 and 0.59 are *fair*, between 0.60 and 0.74 are *good*, and 0.75 and above are *excellent*. Koo and Li also provide guidelines that state ICCs below 0.50 are “poor,” between 0.50 and 0.74 are “fair,” between 0.75 and 0.90 are “good,” and from 0.91 − 1.0 are “excellent.” ICCs were calculated for the full sample. It was hypothesized that TPOCS-RS Family Therapy item- and subscale-level interrater reliability would be at least “fair” (ICC *≥* 0.40; Cicchetti, 1994) for the full sample. See Table 3 for descriptive and interrater reliability information for the TPOCS-RS items and subscales (see Supplemental Table 1 for descriptive information for the TPOCS-RS items and subscales for each group).

**Table 3:** TPOCS-RS item and subscale descriptive data and interrater reliability

Item	Full sample (*N* = 103)					
	*N*	Range	*M (SD)*	ICC	S	K
Family therapy subscale	–	2.80	2.02 (0.70)	0.898	0.23	− 0.93
Targets others	72	5.00	3.08 (1.74)	0.887	0.16	− 1.36
Recruits others	34	2.00	1.29 (0.48)	0.756	1.71	2.41
Parenting style	19	3.00	1.23 (0.61)	0.809	3.33	11.57
Multiparticipant interactions	43	5.00	1.70 (1.14)	0.856	1.96	3.39
Family roles	89	6.00	2.82 (1.35)	0.759	0.54	− 0.06
BPT subscale	–	2.50	1.14 (0.34)	0.848	4.08	22.27
Operant–parent	10	2.00	1.08 (0.29)	0.772	4.78	25.72
Parenting skills	19	4.50	1.19 (0.57)	0.808	5.03	33.28
CBT subscale	–	0.90	1.09 (0.17)	0.912	2.41	6.31
Functional analysis	4	2.50	1.04 (0.15)	0.901	7.88	67.68
Monitoring	5	1.50	1.04 (0.19)	0.750	5.87	37.51
Cognitive distortion	4	1.00	1.03 (0.15)	0.797	5.60	31.76
Relaxation	1	1.50	1.02 (0.15)	0.889	10.15	103.00
Skill building	25	3.50	1.31 (0.67)	0.913	2.57	7.19
Psych subscale	–	1.80	1.34 (0.41)	0.813	1.51	1.97
Transference	3	2.00	1.03 (0.22)	0.761	7.62	62.07
Explores past	49	4.00	1.72 (1.02)	0.789	1.41	1.13
Client resistance	12	3.00	1.12 (0.42)	0.767	4.69	25.89
Interpretation	49	3.50	1.47 (0.66)	0.716	1.84	4.19
CC subscale	–	3.70	3.19 (0.68)	0.764	0.53	0.40
Validates client	102	4.50	2.94 (0.90)	0.650	0.41	− 0.27
Positive regard	95	4.50	2.40 (0.92)	0.655	0.93	1.05
Client perspective	103	4.50	4.21 (1.07)	0.727	− 0.60	−0.09
Not included in subscales	–	–	–	–	–	–
Cognitive education	1	0.50	1.01 (0.05)	NV	10.15	103.00
Coping skills	0	0.00	1.00 (0.00)	NV	–	–
Respondent	0	0.00	1.00 (0.00)	NV	–	–
Operant–child	4	1.00	1.02 (0.13)	0.557	5.87	37.10
Behavioral activation	3	1.00	1.02 (0.12)	NV	6.79	49.16
Modeling	24	2.50	1.24 (0.50)	0.715	2.23	4.86

*Note. TPOCS-RS =* Therapy Process Observational System for Child Psychotherapy-Revised Scale; *N* = the number of times an item was observed as occurring during a session by at least one coder; BPT = behavioral parent training; CBT = cognitive-behavioral therapy; Psych = psychodynamic; CC = client-centered; ICC = intraclass correlation coefficient; S = skewness; K = kurtosis; NV = ICCs not calculated due to lack of variance

Item-level ICCs ranged from 0.56 to 0.91 (*M* ICC = 0.77, *SD* = 0.09), and all but one item displayed at least *good* interrater reliability; *Operant*– *Child* (ICC = 0.56) displayed *fair* reliability. Due to a lack of coder variance, likely because the items were infrequently observed, ICCs could not be calculated for four items (Cognitive Education, Coping Skills, Respondent, and Behavioral Activation). Findings suggest that all TPOCS-RS items that were consistently observed can be coded reliably by independent observers in a sample containing the delivery of family therapy.

To examine the ability of the TPOCS-RS to estimate adherence to family therapy (i.e., convergent validity), a specific RFT subscale was created by selecting TPOCS-RS items that (a) represent the core techniques of family therapy (Hogue et al., 2017, 2019; Hogue et al., 2023); (b) are not a core technique of another treatment modality (i.e., Behavioral Parent Training, CBT, Psychodynamic, or Client-Centered); and (c) had an ICC of at least 0.40 (Cicchetti, 1994). The TPOCS-RS Family Therapy subscale consisted of five items: *Targets Others*, *Recruits Others*, *Parenting Style*, *Multiparticipant Interactions*, and *Family Roles*. The ICC(2,2) value for the TPOCS-RS Family Therapy subscale was 0.90.

Two additional TPOCS-RS subscales were created to assess discriminant validity (i.e., differentiation). First, a subscale was created with the remaining family-focused TPOCS-RS items: *Operant–Parent* and *Parenting Skills*. These two items represent techniques consistent with the behavioral parent training modality (e.g., behavioral reward systems, positive reinforcement, limit-setting), so the subscale was called Behavioral Parent Training (BPT). An item was included in the BPT subscale if it had an ICC(2,2) of at least 0.40 (Cicchetti, 1994). Second, a CBT subscale comprising five TPOCS-RS items considered core CBT techniques for adolescent externalizing problems (Hogue et al., 2020) was created: *Functional Analysis*, *Monitoring*, *Cognitive Distortion*, *Relaxation*, and *Skill Building*. Three items initially considered for inclusion in the CBT subscale were excluded due to low ICC values (*Coping Skills*, *Cognitive Education*, and *Behavioral Activation)*. ICCs for the TPOCS-RS BPT and CBT subscales were all > 0.75.

### Convergent and Discriminant Validity

Convergent validity of the TPOCS-RS Family Therapy subscale, considered evidence for assessing adherence, was evaluated by examining correlations between scores on the observer-rated TPOCS-RS Family Therapy subscale and observer- and clinician-rated scales that assess family therapy (i.e., ITT-ABP FT subscales). Correlation magnitudes were interpreted based on Rosenthal and Rosnow’s (1984) guidelines (correlations are “small” if *r* *≥*.10, “medium” if 0.24 *≤* *r* <.37, and “large” if *r* *≥*.37). Cohen (1988) also provide guidelines that state correlations are small if *r* is 0.1–0.29, medium if *r* is 0.3–0.49, and large if *r* is > 0.5. It was hypothesized that the TPOCS-RS Family Therapy subscale scores would have a large correlation (*r* *≥*.37) with an observer-rated family therapy adherence subscale (i.e., ITT-ABP FT subscale) and a medium correlation (0.24 *≤* *r* <.37) with a clinician-rated subscale of family therapy techniques (i.e., ITT-ABP FT subscale; McLeod et al., 2023). Discriminant validity, evidence supporting differentiation, was evaluated by examining the magnitude and pattern of Pearson product-moment correlations among scores on the TPOCS-RS Family Therapy subscale and scores from (a) observer-rated subscales designed to assess non-family therapy techniques (i.e., TPOCS-RS BPT, CBT, Psychodynamic, Client-Centered subscales; ITT-ABP CBT/MI subscale); (b) subscales of clinician-rated non-family therapy techniques (i.e., ITT-ABP CBT/MI subscale); and (c) an observer-rated scale of client-clinician alliance (i.e., VTAS-R-SF). It was hypothesized that correlations between the TPOCS-RS Family Therapy subscale and all observer-rated subscales of non-family therapy techniques and the alliance would be small-to-medium (*r* <.37). Follow-up contrasts using Fisher r-to-z transformation were calculated for the absolute value of correlations to determine if the correlations produced by the TPOCS-RS Family Therapy subscale followed this pattern. The main analyses were computed in the full sample (*N* = 103); however, since clinicians did not rate the ITT-ABP in the MIP trial, correlations between the TPOCS-RS Family Therapy subscale and the clinician-rated ITT-ABP subscales were computed only in the CASALEAP sample.

As seen in Table 4, correlations in the full sample were large between the TPOCS-RS Family Therapy subscale and the observer-rated ITT-ABP FT subscale (*r* =.660) and the clinician-rated ITT-ABP FT subscale in the CASALEAP sample (*r* =.622). Correlation magnitudes between the TPOCS-RS Family Therapy subscale and the remaining subscales ranged from *r* =.018 to 0.331 and were small to medium in magnitude. Follow-up contrasts revealed that the correlation between the TPOCS-RS Family Therapy subscale and the observer-rated ITT-ABP FT subscale (*r* =.660) was not significantly different from the correlation between the TPOCS-RS Family Therapy subscale and the clinician-rated ITT-ABP FT subscale (*r* =.622; *z* = 0.37, *p* =.711). Additionally, the correlation between the TPOCS-RS Family Therapy subscale and the observer-rated ITT-ABP FT subscale (*r* =.660) was significantly stronger than the correlations between the TPOCS-RS Family Therapy subscale and the (a) TPOCS-RS BPT subscale (*r* =.331; *z* = 3.13, *p* =.002); (b) TPOCS-RS Psychodynamic subscale (*r* =.254; *z* = 3.72, *p* <.001); (c) the TPOCS-RS CBT subscale (*r* =.147; *z* = 4.50, *p <*.001); (d) TPOCS-RS Client-Centered subscale (*r* =.018; *z* = 5.41, *p* <.001); (e) observer-rated ITT-ABP CBT/MI subscale (*r* =.101; *z* = 4.77, *p* <.001); (f) clinician-rated ITT-ABP CBT/MI subscale (*r* =.107; *z* = 3.95, *p* <.01); and (g) VTAS-R-SF (*r* =.194; *z* = 3.52, *p* <.001).

**Table 4:** Correlations between the TPOCS-RS Subscales, ITT-ABP subscales, and VTAS-R-SF

	ORITT-ABP FT	CRITT-ABP FT	TPOCS- RS BPT	TPOCS-RS CBT	TPOCS- RS Psych	TPOCS-RS CC	ORITT-ABP CBT/MI	CRITT-ABP CBT/MI	VTAS-R-SF
TPOCS-RS FT	0.660** (*N* = 98)	0.622** (*N* = 54)	0.331** (*N* = 103)	− 0.147 (*N* = 103)	0.254 (*N* = 103)	0.018 (*N* = 103)	− 0.101 (*N* = 98)	0.107 (*N* = 54)	− 0.194 (*N* = 58)
OR ITT-ABP FT	–	0.620** (*N* = 50)	0.257* (*N* = 98)	0.002 (*N* = 98)	0.309* (*N* = 98)	0.022 (*N* = 98)	0.087 (*N* = 98)	0.251 (*N* = 50)	− 0.028 (*N* = 58)
CR ITT-ABP FT	–	–	0.340* (*N* = 54)	− 0.272* (*N* = 54)	0.127 (*N* = 54)	− 0.215 (*N* = 54)	− 0.050 (*N* = 50)	0.595** (*N* = 54)	− 0.097 (*N* = 39)
TPOCS-RS BPT	–	–	–	− 0.118 (*N* = 103)	− 0.045 (*N* = 103)	− 0.285* (*N* = 103)	− 0.103 (*N* = 98)	0.095 (*N* = 54)	0.113 (*N* = 58)
TPOCS-RS CBT	–	–	–	–	0.115 (*N* = 103)	0.332** (*N* = 103)	0.305* (*N* = 98)	− 0.007 (*N* = 54)	− 0.084 (*N* = 58)
TPOCS-RS Psych	–	–	–	–	–	0.180 (*N* = 103)	0.124 (*N* = 98)	0.061 (*N* = 54)	− 0.092 (*N* = 58)
TPOCS-RS CC	–	–	–	–	–	–	0.199* (*N* = 98)	− 0.138 (*N* = 54)	0.160 (*N* = 58)
OR ITT-ABP CBT/MI	–	–	–	–	–	–	–	0.140 (*N* = 50)	0.304* (*N* = 58)
CR ITT-ABP CBT/MI	–	–	–	–	–	–	–	–	− 0.088 (*N* = 39)

*Note. TPOCS-RS* = Therapy Process Observational System for Child Psychotherapy-Revised Scale; *ITT-ABP* = Inventory of Therapeutic Techniques for Adolescent Behavior Problems; *VTAS-R-SF* = Vanderbilt Therapeutic Alliance Scale Revised Short Form; OR = observer-rated; CR = clinician-rated; FT = family therapy; BPT = behavioral parent training; CBT = cognitive-behavioral therapy; MI = motivational interviewing; Psych = psychodynamic; CC = client-centered

* *p* <.05, ** *p* <.001

A similar pattern of correlations was observed when examined in the family therapy groups (RFT and MIP) only. As seen in Supplemental Table 2, the correlation between the TPOCS-RS Family Therapy subscale and the observer-rated ITT-ABP FT subscale (*r* =.563) was large, and the correlation between the TPOCS-RS Family Therapy subscale and the clinician-rated ITT-ABP FT subscale (*r* =.314) was medium. Correlations with the TPOCS-RS Family Therapy subscale and the remaining subscales ranged from *r* =.032 to 0.296 and were small to medium in magnitude (small when using Cohen’s (1988) guidelines). Follow-up contrasts revealed that the correlation between the TPOCS-RS Family Therapy subscale and the observer-rated ITT-ABP FT subscale (*r* =.563) was not significantly different from the correlation between the TPOCS-RS Family Therapy subscale and the clinician-rated ITT-ABP FT subscale (*r* =.314; *z* = 1.49, *p* =.136), or the VTAS-R-SF (*r* =.296, *z* = 1.69, *p* =.091). The correlation between the TPOCS-RS Family Therapy subscale and the observer-rated ITT-ABP FT subscale (*r* =.563) was significantly stronger than the correlations between the TPOCS-RS Family Therapy subscale and the (a) TPOCS-RS BPT subscale (*r* =.294; *z* = 1.11, *p* =.035); (b) TPOCS-RS Psychodynamic subscale (*r* =.214; *z* = 2.65, *p* =.008); (c) the TPOCS-RS CBT subscale (*r* =.077; *z* = 3.53, *p <*.001); (d) TPOCS-RS Client-Centered subscale (*r* =.032; *z* = 3.81, *p* <.001); (e) observer-rated ITT-ABP CBT/MI subscale (*r* =.230; *z* = 2.52, *p* =.012); and (f) the clinician-rated ITT-ABP CBT/MI subscale (*r* =.198; *z* = 2.08, *p* =.038).

Together, these results indicate that the TPOCS-RS Family Therapy subscale scores are more closely related to scores on measures of the same construct (convergent validity) than to subscale scores of distinct constructs (discriminant). Moreover, the pattern of correlations produced by the TPOCS-RS Family Therapy subscale mirrors the pattern produced by the observer-rated ITT-ABP FT subscale. These results are consistent with hypotheses, supportive of convergent and discriminant validity, and provide evidence for using the TPOCS-RS Family Therapy subscale for assessing adherence to and differentiation from family therapy.

### Discriminative Validity

To explore discriminative validity, one-way ANOVAs were conducted to examine group differences in TPOCS-RS Family Therapy subscale scores, followed by pairwise comparisons using estimated marginal means to account for the nested data design (sessions nested within clients nested within clinicians; Barber et al., 2004) with a Bonferroni adjustment (*p* <.017). For discriminative validity analyses only, the TPOCS-RS Family Therapy subscale scores were derived from the highest item score on the subscale for each session, consistent with previous research exploring the ability of the TPOCS-RS subscale scores to identify group differences (e.g., Smith et al., 2017). Because not all techniques on a subscale would be expected to be delivered in a single session, averaging all items included in a subscale may underestimate the dosage and subsequently underestimate group differences (see Smith et al., 2017). It was hypothesized that TPOCS-RS Family Therapy subscale scores would be (a) significantly higher for RFT than for UC, (b) significantly higher for RFT than for MIP, and (c) significantly higher for MIP than UC.

Results of a one-way ANOVA demonstrated that TPOCS-RS Family Therapy subscale scores significantly differed between groups (*F*[2,100] = 28.69, *p* <.001). Pairwise comparisons revealed significantly higher TPOCS-RS Family Therapy subscale scores in RFT (*M* = 4.81, *SE* = 0.21) than in MIP (*M* = 3.47, *SE* = 0.18, 95% CI [0.67,2.02], *p* <.001) or UC (*M* = 2.18, *SE* = 0.29, 95% CI [1.76, 3.50] *p* <.001). TPOCS-RS Family Therapy scores were also significantly higher for MIP (*M* = 3.47, *SE* = 0.18) than for UC (*M* = 2.18, *SE* = 0.29, 95% CI [0.46, 2.11], *p* <.001). Taken together, scores on the TPOCS-RS Family Therapy subscale were highest for RFT, second highest for MIP, and lowest for UC. The observed group differences in TPOCS-RS Family Therapy subscale scores are consistent with hypothesized group differences and supportive of discriminative validity.

### Treatment Differentiation

One-way ANOVAs were conducted to examine group differences in the TPOCS-RS BPT, CBT, Client-Centered, and Psychodynamic subscale scores, followed by pairwise comparisons using estimated marginal means to examine treatment differentiation. A Bonferroni adjustment was applied for the TPOCS-RS subscale group (*p* <.0125) and pairwise comparisons (*p* <.017). Per above, subscale scores were calculated using the highest item score on the subscale. We hypothesized that the TPOCS-RS subscale scores would (a) evidence no significant differences between groups and (b) be relatively low for techniques non-prescribed by family therapy (i.e., BPT, CBT, Psychodynamic). As seen in Table 5, scores across the three groups on the TPOCS-RS BPT, CBT, and Psychodynamic subscales were low (< 3) and moderate (between 3 and 6) on the Client-Centered subscale (McLeod et al., 2015). After a Bonferroni correction, there was a significant effect of group for the TPOCS-RS BPT subscale scores (*F*[2,100] = 5.65, *p* =.005) and the TPOCS-RS Psychodynamic subscale scores (*F*[2,100] = 5.13, *p* =.008). Pairwise comparisons for the TPOCS-RS BPT subscale revealed that scores were significantly higher in RFT (*M* = 1.50, *SE* = 0.10) than in MIP (*M* = 1.13, *SE* = 0.08, 95% CI [0.06, 0.68], *p* =.015) or UC (*M* = 1.03, *SE* = 0.13, 95% CI [0.07, 0.88], *p* =.015). The TPOCS-RS Psychodynamic subscale scores were significantly higher in RFT (*M =* 2.36, *SE* = 0.17) than in UC (*M* = 1.53, *SE* = 0.23, 95% CI [0.14, 1.52], *p* =.012). No significant group differences were found in the TPOCS-RS Client-Centered subscale scores (*F*[2,100] = 1.72, *p* =.185) or TPOCS-RS CBT subscale scores (*F*[2,100] = 3.14, *p* =.047).

**Table 5:** One-way ANOVA with pairwise comparison of estimated marginal means by treatment group

	Adjusted M (SE) *(N* = 103)			One-way ANOVA		Pairwise Comparisons					
						RFT x MIP		RFT x UC		MIP x UC	
	RFT	MIP	UC	*F*(2,100)	*p*	95% CI	*p*	95% CI	*p*	95% CI	*p*
TPOCS-RS FT	4.81 (0.21)	3.47 (0.18)	2.18 (0.29)	28.69	< 0.001*	0.67, 2.02	< 0.001*	1.76, 3.50	< 0.001*	0.46, 2.11	< 0.001*
TPOCS-RS BPT	1.50 (0.10)	1.13 (0.08)	1.03 (0.13)	5.65	0.005*	0.06, 0.68	0.015*	0.07, 0.88	0.015*	− 0.28, 0.49	1.00
TPOCS-RS CBT	1.50 (0.12)	1.21 (0.10)	1.63 (0.16)	3.14	0.047	− 0.09, 0.66	0.200	− 0.61, 0.35	1.00	− 0.88, 0.04	0.086
TPOCS-RS Psych	2.36 (0.17)	1.82 (0.14)	1.53 (0.23)	5.13	0.008*	0.01, 1.08	0.046	0.14, 1.52	0.012*	− 0.36, 0.94	0.846
TPOCS-RS CC	4.20 (0.15)	4.53 (0.13)	4.21 (0.21)	1.72	0.185	− 0.63, 0.61	1.00	− 0.81, 0.15	0.294	− 0.27, 0.91	0.565

Note. *TPOCS-RS* = Therapy Process Observational System for Child Psychotherapy-Revised Scale; FT = family therapy; BPT = behavioral parent training; CBT = cognitive-behavioral therapy; Psych = Psychodynamic; RFT = routine family therapy; MIP = medication integration protocol; UC = usual clinical care

*Significant after Bonferroni adjusted *p*-value

In sum, RFT had significantly higher scores on two subscales (BPT and Psychodynamic), and scores on the TPOCS-RS BPT, CBT, and Psychodynamic subscales were consistently low across groups.

## Discussion

The present study aimed to determine if the TPOCS-RS Family Therapy subscale scores demonstrate evidence of reliability and validity to ascertain the potential of using the subscale to assess adherence to and differentiation from family therapy. This study expands on prior research findings that the TPOCS-RS can estimate adherence to and differentiation from the CBT modality in samples of youth with anxiety (McLeod et al., 2015, 2022). Interrater reliability was acceptable for the TPOCS-RS Family Therapy items when used to code treatment sessions containing family therapy. Moreover, the study findings supported the construct validity of the TPOCS-RS Family Therapy and ITT-ABP subscales. Findings also indicated that (a) the TPOCS-RS Family Therapy subscale can detect known differences between clinicians delivering family therapy and clinicians who do not, and (b) the remaining TPOCS-RS subscales can be used to assess family therapy differentiation from other treatment modalities in an effectiveness trial. Together, these findings support the convergent and discriminant validity of the Family Therapy subscale and indicate that this subscale may be able to estimate adherence to and differentiation from family therapy.

All TPOCS-RS family therapy items in this study achieved good to excellent interrater reliability (Cicchetti, 1994). The average interrater reliability of the TPOCS-RS items (*M* ICC = 0.77) was consistent with the mean TPOCS-RS item-level interrater reliability in past research (*M* ICC range = 0.61 to 0.84; e.g., Herschell et al., 2020; McLeod et al., 2015; McLeod et al., 2022). Subscale-level interrater reliability was also *excellent* and consistent with prior research (ICC range = 0.72 to 0.94, *M* ICC = 0.86; McLeod et al., 2015; McLeod et al., 2022). These findings add to existing evidence suggesting that independent observers can reliably code the TPOCS-RS items for different youth emotional and behavioral problems (Herschell et al., 2020; McLeod et al., 2015) and that reliability evidence extends to sessions of family therapy for adolescent externalizing problems conducted in community mental health centers.

The magnitude and pattern of correlations observed in the current study support the convergent validity of TPOCS-RS Family Therapy subscale scores. As hypothesized, the TPOCS-RS Family Therapy subscale scores demonstrated convergent validity via large correlations with scores from observer- and clinician-rated measures designed to capture techniques found in family therapy. Moreover, the pattern of correlations produced by the TPOCS-RS Family Therapy subscale scores mirrored those of the observer-rated measure designed to capture family therapy techniques, supporting the construct validity of both measures. Although the TPOCS-RS was not originally designed to assess adherence to family therapy techniques, the similarity in correlation patterns among scores produced by the TPOCS-RS subscales and scores produced by the ITT-ABP FT suggests that TPOCS-RS subscale scores may be helpful for this purpose.

Our findings also provide evidence of discriminant validity. Specifically, the magnitude and pattern of the correlations between the TPOCS-RS Family Therapy subscale, BPT techniques, and other non-family therapy techniques, and the client-clinician alliance are mainly consistent with hypotheses and supportive of discriminant validity. Moreover, these patterns are aligned with those produced by observational treatment integrity measures designed to assess different therapy modalities, with evidence of discriminant validity supported by low to medium correlations with alternate therapy domains and the alliance (e.g., Carroll et al., 2000; Hogue et al., 2008; McLeod et al., 2015). These findings support using the Family Therapy subscale to assess differentiation from family therapy in that scores on the Family Therapy subscale appear distinct from scores on other modality-based domains. These findings also add to previous research indicating that the other TPOCS-RS subscales are distinct, thus supporting using these subscales collectively to assess differentiation (see McLeod et al., 2015; McLeod et al., 2022). With these findings, evidence supports the convergent and discriminant validity of the TPOCS-RS Family Therapy subscale.

The TPOCS-RS Family Therapy subscale differentiated between groups expected to differ in their delivery of family therapy techniques. Group differences in TPOCS-RS Family Therapy subscale scores were highest for the RFT group, second highest for MIP, and lowest for UC, consistent with hypotheses. These findings are aligned with previous research demonstrating the same pattern of scores from the ITT-ABP FT subscale (Hogue et al., 2014b, 2016). These findings support the discriminative validity of the TPOCS-RS Family Therapy subscale, indicating that the Family Therapy subscale may be used to conduct manipulation checks between family therapy and other treatment groups, such as usual clinical care, a key element of interpreting results from effectiveness trials (McLeod et al., 2022).

By providing evidence supporting the convergent and discriminant validity of the Family subscale, this study addresses a limitation of prior research with the TPOCS-RS. Previously, when used to code treatment sessions of samples delivering manual-guided treatment, the TPOCS-RS has focused on CBT (e.g., McLeod et al., 2022; Smith et al., 2017) and BPT (Wood et al., 2006). Though evidence supports the use of the non-CBT subscales for assessing differentiation (i.e., subscale scores demonstrate discriminant validity; McLeod et al., 2015), the evidence did not support that items on these subscales mapped onto evidence-based programs. With this study, evidence now suggests that the TPOCS-RS subscales can be used to assess adherence to CBT, BPT, and family therapy, thus broadening the applications of the TPOCS-RS within effectiveness research.

Our findings indicate that a single measure, the TPOCS-RS, may be able to assess adherence to and differentiation from the family therapy modality. The TPOCS-RS subscale scores that assess techniques non-prescribed by family therapy (i.e., CBT, BPT, Psychodynamic) were low in magnitude (< 3) and consistent across the three groups. This pattern indicates that treatment differentiation was achieved across the groups and is similar to what has been observed when differentiation was assessed in other effectiveness trials (see McLeod et al., 2015). In contrast, scores on the TPOCS-RS Client-Centered subscale were moderate in magnitude and did not differ across groups. This suggests that clinicians delivered a moderate dose of client-centered techniques, consistent with previous research suggesting that these techniques are consistently delivered in community settings (Herschell et al., 2020; Smith et al., 2017). Overall, these findings suggest that the TPOCS-RS subscales were able to assess treatment differentiation within the current sample.

Though research has generally found low to moderate concordance between observer and clinician treatment fidelity ratings (McLeod et al., 2023), we found medium to large correlations (*r*s 0.31 to 0.62; see Table 4 and Supplemental Table 2) between the TPOCS-RS and clinician-rated adherence ratings, consistent with past research comparing the clinician and independent observer ratings of the ITT-ABP (Hogue et al., 2015b, 2022). In addition to supporting the construct validity of the observer-rated subscales, these findings support the reliability of the clinician-rated ITT-ABP FT subscale. Given the cost of using observer-rated treatment integrity measures, it is noteworthy that this clinician-rated subscale demonstrates such strong concordance with observer-rated scales. Looking forward, these findings may help pave the way for more cost-effective, feasible treatment integrity measures that can be incorporated into the workflow of community mental health centers.

The present study has several limitations. First, MIP sessions (47.6%) did not include clinician-rated treatment integrity data and had some missing alliance data; therefore, validity analyses involving those constructs were conducted with a smaller sample. Additionally, while several elements of the TPOCS-RS make it a valuable tool for effectiveness research, it is costly in terms of time and resources. Community mental health settings are unlikely to have the financial resources to hire independent coders or the ability to pay staff to double-code each session to achieve the interrater reliability seen in the present study. Double-coding a portion of the sample would be less costly (e.g., 20%), but this would likely result in lower ICC estimates (Hallgren, 2012) and still may be too costly for community settings. Thus, as research progresses, more cost-effective treatment integrity measures are needed to support efforts to implement and sustain evidence-based programs in community mental health centers.

Overall, findings support the ability of the TPOCS-RS to estimate adherence to and differentiation from the family therapy modality. The present study adds to previous research findings that the TPOCS-RS items can be combined to assess treatment adherence (McLeod et al., 2022) and differentiation (McLeod et al., 2015, 2022) by being the first to explore its ability to do so in a sample of adolescents receiving family therapy or usual care for externalizing problems. Notably, in the current study and past research (McLeod et al., 2022), the TPOCS-RS has been used to assess differentiation by comparing a treatment modality (e.g., family therapy, CBT) to usual clinical care. Future research should explore the ability of the TPOCS-RS to assess differentiation when used to assess multiple specific treatment modalities. A single non-problem, non-treatment specific measure that can assess both adherence and differentiation can serve as a more efficient tool for identifying what, and to what extent, treatment techniques were delivered in an effectiveness trial, aiding in the interpretation of observed or non-observed group differences (McLeod et al., 2022). Looking forward, a single generic measure that can assess adherence and differentiation, like the TPOCS-RS, may be helpful in quality improvement efforts in the mental health field (McLeod et al., 2013).

## Electronic Supplementary Material

Below is the link to the electronic supplementary material.

Supplementary Material 1
