# Supplementary material for: Using a Single Measure To Assess Adherence and Differentiation in Family Therapy for Adolescent Externalizing Problems
Source: Adm Policy Ment Health. 2025 Apr 28;52(4):687–700. doi: 10.1007/s10488-025-01445-y (PMC12310758; doi:10.1007/s10488-025-01445-y)
Supplement: Supplementary file 1 — Supplementary Material 1 [file 10488_2025_1445_MOESM1_ESM.docx]

| **Table S1**  *TPOCS-RS Item and Subscale Descriptive Data and Interrater Reliability by Group* | | | | | | | | | | | | | | | | | | |
| --- | --- | --- | --- | --- | --- | --- | --- | --- | --- | --- | --- | --- | --- | --- | --- | --- | --- | --- |
| Item | Routine Family Therapy (*n* = 35) | | | | | | Usual Care (*n* = 19) | | | | | | MIP (*n* = 49) | | | | | |
|  | *N* | Range | *M(SD)* | ICC | S | K | *N* | Range | *M(SD)* | ICC | S | K | *N* | Range | *M(SD)* | ICC | S | K |
| *Family Therapy Subscale* | - | 2.5 | 2.53 (0.60) | .805 | -0.24 | -0.21 | - | 1.7 | 1.37 (0.48) | .879 | 1.74 | 2.73 | - | 2.1 | 1.91 (0.57) | .892 | 0.21 | -1.01 |
| Targets Others | 33 | 5.0 | 4.04 (1.52) | .731 | -0.33 | -0.86 | 3 | 3.0 | 1.40 (0.95) | .985 | 2.18 | 3.32 | 36 | 5.0 | 3.04 (1.64) | .896 | 0.09 | -1.41 |
| Recruits Others | 15 | 2.0 | 1.37 (0.80) | .753 | 1.48 | 1.61 | 6 | 1.0 | 1.24 (0.39) | .353 | 1.31 | 0.17 | 13 | 2.0 | 1.24 (0.47) | .858 | 2.00 | 3.67 |
| Parenting Style | 10 | 3.0 | 1.37 (0.80) | .758 | 2.52 | 5.77 | 1 | 1.0 | 1.05 (0.23) | 1.00 | 4.36 | 19.00 | 8 | 3.0 | 1.19 (0.54) | .883 | 3.65 | 15.55 |
| Multiparticipant Interactions | 18 | 5.0 | 2.09 (1.48) | .927 | 1.36 | 0.78 | 2 | 2.5 | 1.21 (0.65) | .680 | 3.13 | 9.41 | 23 | 4.0 | 1.60 (0.92) | .734 | 2.02 | 4.27 |
| Family Roles | 33 | 5.5 | 3.79 (1.31) | .714 | 0.36 | -0.09 | 12 | 3.0 | 1.95 (1.07) | .510 | 0.88 | -0.59 | 42 | 4.0 | 2.47 (1.05) | .775 | 0.25 | -0.87 |
| *BPT Subscale* | - | 2.5 | 1.29 (0.49) | .835 | 2.94 | 11.36 | - | 0.3 | 1.01 (0.06) | NV | 4.36 | 19.00 | - | 1.3 | 1.08 (0.23) | .881 | 3.73 | 15.31 |
| Operant–Parent | 6 | 1.5 | 1.13 (0.33) | .677 | 2.98 | 9.35 | 0 | 0.0 | 1.00 (0.00) | NV | - | - | 4 | 2.0 | 1.07 (0.31) | .844 | 5.59 | 34.24 |
| Parenting Skills | 13 | 4.5 | 1.44 (0.86) | .816 | 3.34 | 14.09 | 1 | 0.5 | 1.03 (0.22) | NV | 4.36 | 19.00 | 5 | 1.5 | 1.08 (0.28) | .728 | 3.92 | 16.36 |
| *CBT Subscale* | - | 0.6 | 1.11 (0.18) | .897 | 1.84 | 2.56 | - | 0.9 | 1.15 (0.23) | .928 | 2.17 | 5.27 | - | 0.5 | 1.05 (0.12) | .901 | 2.66 | 6.80 |
| Functional Analysis | 3 | 2.5 | 1.11 (0.46) | .933 | 4.73 | 23.74 | 0 | 0.0 | 1.00 (0.00) | NV | - | - | 1 | 0.5 | 1.01 (0.07) | NV | 7.00 | 49.00 |
| Monitoring | 0 | 0.0 | 1.00 (0.00) | NV | - | - | 2 | 0.5 | 1.05 (0.00) | NV | 2.80 | 6.51 | 3 | 1.5 | 1.06 (0.26) | .855 | 4.64 | 22.03 |
| Cognitive Distortion | 1 | 0.5 | 1.01 (0.09) | NV | 5.92 | 35.00 | 1 | 1.0 | 1.05 (0.23) | 1.00 | 4.36 | 19.00 | 2 | 1.0 | 1.03 (0.16) | .797 | 5.54 | 31.76 |
| Relaxation | 0 | 0.0 | 1.00 (0.00) | NV | - | - | 1 | 1.5 | 1.08 (0.34) | .889 | 0.34 | 19.00 | 0 | 0.0 | 1.00 (0.00) | NV | - | - |
| Skill Building | 11 | 3.0 | 1.40 (0.74) | .905 | 2.07 | 4.07 | 7 | 3.5 | 1.55 (0.93) | .920 | 2.06 | 4.76 | 7 | 2.0 | 1.15 (0.42) | .913 | 3.01 | 8.95 |
| *Psychodynamic Subscale* | - | 1.8 | 1.54 (0.53) | .823 | 0.86 | -0.44 | - | 0.9 | 1.18 (0.25) | .700 | 1.56 | 2.02 | - | 1.0 | 1.25 (0.28) | .724 | 0.96 | -0.05 |
| Transference | 3 | 2.0 | 1.10 (0.38) | .758 | 4.35 | 19.89 | 0 | 0.0 | 1.00 (0.00) | NV | - | - | 0 | 0.0 | 1.00 (0.00) | NV | - | - |
| Explores Past | 20 | 4.0 | 1.97 (1.18) | .823 | 1.08 | 0.08 | 8 | 2.0 | 1.40 (0.59) | .485 | 1.58 | 1.94 | 21 | 3.5 | 1.67 (0.99) | .778 | 1.43 | 1.14 |
| Client Resistance | 7 | 3.0 | 1.23 (0.62) | .792 | 3.50 | 12.92 | 2 | 1.0 | 1.11 (0.32) | 1.00 | 2.80 | 6.51 | 3 | 1.0 | 1.05 (0.21) | .413 | 4.20 | 16.88 |
| Interpretation | 23 | 3.5 | 1.84 (0.87) | .762 | 1.04 | 0.96 | 4 | 1.5 | 1.24 (0.51) | .843 | 2.01 | 2.71 | 22 | 1.0 | 1.29 (0.35) | .085 | 0.84 | -0.51 |
| *Client-Centered Subscale* | - | 3.7 | 3.13 (0.82) | .808 | 0.57 | 0.39 | - | 2.5 | 3.26 (0.75) | .715 | 0.74 | -0.50 | - | 2.5 | 3.20 (0.54) | .778 | 0.39 | 0.024 |
| Validates Client | 34 | 4.5 | 3.17 (1.02) | .602 | 0.23 | -0.15 | 19 | 3.0 | 2.84 (0.85) | .642 | 0.18 | 0.26 | 49 | 3.0 | 2.82 (0.80) | .727 | 0.45 | -0.76 |
| Positive Regard | 32 | 4.5 | 2.33 (1.02) | .741 | 1.33 | 1.97 | 19 | 3.5 | 2.82 (0.95) | .578 | 0.71 | 0.26 | 44 | 3.5 | 2.30 (0.80) | .655 | 0.58 | 0.56 |
| Client Perspective | 35 | 4.5 | 3.89 (1.24) | .760 | -0.24 | -0.69 | 19 | 3.0 | 4.13 (1.03) | .634 | -0.44 | -1.14 | 49 | 4.5 | 4.48 (0.90) | .690 | -0.81 | 1.45 |
| *Not Included in Subscales* | - | - | - | - | - | - | - | - | - | - | - | - | - | - | - | - | - | - |
| Cognitive Ed | 0 | 0.0 | 1.00 (0.00) | NV | - | - | 0 | 0.0 | 1.00 (0.00) | NV | - | - | 1 | 0.5 | 1.01 (0.07) | NV | 7.00 | 49.00 |
| Coping Skills | 0 | 0.0 | 1.00 (0.00) | NV | - | - | 0 | 0.0 | 1.00 (0.00) | NV | - | - | 0 | 0.0 | 1.00 (0.00) | NV | - | - |
| Respondent | 0 | 0.0 | 1.00 (0.00) | NV | - | - | 0 | 0.0 | 1.00 (0.00) | NV | - | - | 0 | 0.0 | 1.00 (0.00) | NV | - | - |
| Operant – Child | 3 | 1.0 | 1.06 (0.20) | .660 | 3.81 | 15.05 | 1 | 0.5 | 1.03 (0.12) | NV | 4.36 | 0.52 | 0 | 0.0 | 1.00 (0.00) | NV | - | - |
| Behavioral Activation | 1 | 1.0 | 1.03 (0.17) | NV | 5.92 | 35.00 | 0 | 0.0 | 1.00 (0.00) | NV | - | - | 2 | 0.5 | 1.02 (0.10) | NV | 4.79 | 21.83 |
| Modeling | 16 | 2.5 | 1.53 (0.69) | .678 | 1.13 | 0.59 | 4 | 1.0 | 1.13 (0.28) | .481 | 2.16 | 4.25 | 4 | 1.5 | 1.08 (0.30) | .880 | 3.77 | 13.99 |

Note. TPOCS-RS = Therapy Process Observational System for Child Psychotherapy-Revised Scale; *N* = the number of times an item was observed as occurring during a session by at least one coder; ICC = intraclass correlation coefficient; S = skewness; K = kurtosis; NV = ICCs not calculated due to lack of variance.

**Table S2**

*Correlations Between the TPOCS-RS Subscales, ITT-ABP subscales, and VTAS-R-SF in the FT and MIP groups only*

|  | OR ITT-ABP FT | CR ITT-ABP FT | TPOCS-  RS BPT | TPOCS-RS CBT | TPOCS-  RS Psych | TPOCS-RS CC | OR ITT-ABP CBT/MI | CR ITT-ABP CBT/MI | VTAS-R-SF |
| --- | --- | --- | --- | --- | --- | --- | --- | --- | --- |
| TPOCS-RS FT | .563**  (*N* = 81) | .314  (*N* = 35) | .294**  (*N* = 84) | -.077  (*N* = 84) | .214  (*N* = 84) | .032  (*N* = 84) | -.230*  (*N* = 81) | -.198  (*N* = 35) | -.296  (*N* = 42) |
| OR ITT-ABP FT | - | .346*  (*N* = 33) | .231*  (*N* = 81) | .063  (*N* = 81) | .328**  (*N* = 81) | -.056  (*N* = 81) | -.034  (*N* = 81) | .129  (*N* = 33) | -.092  (*N* = 42) |
| CR ITT-ABP FT | - | - | .244  (*N* = 35) | -.354*  (*N* = 35) | -.143  (*N* = 35) | -.367*  (*N* = 35) | -.405*  (*N* = 33) | .536**  (*N* = 35) | -.110  (*N* = 23) |
| TPOCS-RS BPT | - | - | - | -.107  (*N* = 84) | -.078  (*N* = 84) | -.323**  (*N* = 84) | -.130  (*N* = 81) | .017  (*N* = 35) | .106  (*N* = 42) |
| TPOCS-RS CBT | - | - | - | - | .231*  (*N* = 84) | .331**  (*N* = 84) | .282*  (*N* = 81) | -.158  (*N* = 35) | -.046  (*N* = 42) |
| TPOCS-RS Psych | - | - | - | - | - | .214  (*N* = 84) | .127  (*N* = 81) | -.068  (*N* = 35) | -.051  (*N* = 42) |
| TPOCS-RS CC | - | - | - | - | - | - | .141  (*N* = 81) | -.210  (*N* = 35) | .087  (*N* = 42) |
| OR ITT-ABP CBT/MI | - | - | - | - | - | - | - | -.038  (*N* = 33) | .400**  (*N* = 42) |
| CR ITT-ABP CBT/MI | - | - | - | - | - | - | - | - | .175  (*N* = 23) |

*Note. TPOCS-RS* = Therapy Process Observational System for Child Psychotherapy-Revised Scale*; ITT-ABP* = Inventory of Therapeutic Techniques for Adolescent Behavior Problems; *VTAS-R-SF* = Vanderbilt Therapeutic Alliance Scale Revised Short Form; OR = observer-rated; CR = clinician-rated; FT = family therapy; BPT = behavioral parent training; CBT = cognitive-behavioral therapy; MI = motivational interviewing; Psych = psychodynamic; CC = client-centered.

* *p* < .05, ** *p* < .001
